# Improving Yield and Yield Stability in Winter Rye by Hybrid Breeding

**DOI:** 10.3390/plants11192666

**Published:** 2022-10-10

**Authors:** Bernd Hackauf, Dörthe Siekmann, Franz Joachim Fromme

**Affiliations:** 1 Julius Kühn Institute, Institute for Breeding Research on Agricultural Crops, Rudolf-Schick-Platz 3a, 18190 Sanitz, Germany; 2 Hybro Saatzucht GmbH & Co. KG, Langlinger Straße 3, 29565 Wriedel, Germany; 3 Hybro Saatzucht GmbH & Co. KG, Kleptow 53, 17291 Schenkenberg, Germany

**Keywords:** *Secale cereale*, reciprocal recurrent selection, genetic resources, CMS, fertility restoration, ergot defense, linkage drag, SMART breeding, gibberellin, semidwarf, ideotype, lodging resistance, crop diversification, sustainable bioeconomy, European Green Deal

## Abstract

Rye is the only cross-pollinating small-grain cereal. The unique reproduction biology results in an exceptional complexity concerning genetic improvement of rye by breeding. Rye is a close relative of wheat and has a strong adaptation potential that refers to its mating system, making this overlooked cereal readily adjustable to a changing environment. Rye breeding addresses the emerging challenges of food security associated with climate change. The systematic identification, management, and use of its valuable natural diversity became a feasible option in outbreeding rye only following the establishment of hybrid breeding late in the 20th century. In this article, we review the most recent technological advances to improve yield and yield stability in winter rye. Based on recently released reference genome sequences, SMART breeding approaches are described to counterbalance undesired linkage drag effects of major restorer genes on grain yield. We present the development of gibberellin-sensitive semidwarf hybrids as a novel plant breeding innovation based on an approach that is different from current methods of increasing productivity in rye and wheat. Breeding of new rye cultivars with improved performance and resilience is indispensable for a renaissance of this healthy minor cereal as a homogeneous commodity with cultural relevance in Europe that allows for comparatively smooth but substantial complementation of wheat with rye-based diets, supporting the necessary restoration of the balance between human action and nature.

## 1. Introduction

Rye (*Secale cereale* L.) entered its European growth habitat as a weedy grass in wheat/barley agricultural systems and evolved as a secondary crop by initially mimicking the visual appearance of *Triticum* spp. and *Hordeum vulgare* [1]. Cultivation of rye became most intensive in the high Middle Ages [2], and rye as a staple food contributed substantially to the survival of post-Roman societies in Europe [3]. The cultivation of rye as a standout crop is discussed as a central parameter that triggered Europe’s unique industrial rise and Europe’s position in shaping the world [4]. Today, rye is still integrated in grain production systems, mainly within a belt that ranges from the North German Plain through Poland, Ukraine, Belarus, Scandinavia, and the Baltics into central and northern Russia. Indeed, rye contributed—with less than 3.3%—to the 285.3 million tonnes of cereal grain produced in 2020 [5] in the European Union (EU), the main rye- producing area globally. Grain yield potential is currently the main driver of rye cultivation for the prevailing market, but this healthy grain offers further important features supporting the sustainable production of food and feed which cannot be considered in this review article.

Partial life cycle assessment and mixed model analyses recently identified a ~20% lower greenhouse gas emission and a ~8% lower carbon footprint for rye as compared to wheat production [6]. An increase in rye production, thus, offers a viable option for the European Green Deal to transform the EU into a sustainable, resource-efficient, and competitive bioeconomy that ensures no net emissions of greenhouse gases by 2050. Improved breeding efforts are strategically important for enhancing the competitiveness of rye in modern agricultural production systems. In rye, the first hybrid cultivars were released less than four decades ago and heralded a turning point concerning the genetic improvement of this outbreeding small-grain cereal in particular with respect to grain yield, the most crucial target trait in rye improvement programs [7]. Here, we review most recent technological advances to further improve yield and yield stability in winter rye. We introduce the development of gibberellin-sensitive semidwarf hybrids as a novel strategy to increase productivity that is different from current methods in the genetic improvement of rye and wheat.

## 2. Rye Has Strong Potential for Adaptation to a Changing Climate

In recent decades, European agriculture has experienced higher, more pronounced and more widespread climate variability as compared to the long-term average [8]. In 2022, 47% of Europe received less precipitation than usual, resulting in soil moisture deficit, and 17% of EU vegetation and crops showed the negative effects of drought according to data released by the European Drought Observatory [9]. This threat of climate change to productivity caused short-term fluctuations in food production and put human activities under pressure with a resulting need to design new adaptation and mitigation strategies. Europe counts among the major producers of wheat (*Triticum aestivum*), the staple crop for an estimated 35% of the human population, which will continue to play a critical role to ensure an adequate and affordable intake of calories and proteins in diets until 2050 [10]. Rye diverged from *T. aestivum* approximately 3–4 million years ago [11] and is currently in a stand-by modus for the redesign of the European food system, which is claimed in the EU Farm to Fork Strategy. Tackling the challenges of food security under climate change requires a paradigm shift from the current incremental adaptation strategies towards transformative alternatives that also place an equal emphasis on human nutrition and health, as well as environmental sustainability [12,13]. The renaissance of rye as a homogeneous commodity with its cultural relevance in Europe allows for comparatively smooth but substantial complementation of wheat with rye-based diets, supporting the necessary restoration of the balance between human action and nature. Furthermore, the diversification of cereal production with rye counterbalances the declining crop productivity and yields of staple crops impaired by weather variability and climate change and thereby serves as an insurance for food security. Particularly under the harsh European environments north of the alps with strong winters and poor, podsolic soils, early on, the cross-pollinating winter rye outperformed the more sensitive self-fertilizing wheat and barley [2]. Indeed, rye’s potential to contribute to sustainable and secure food systems under climate change persists in the 21st century. A comprehensive analysis of trends in the mean and variance of winter wheat and winter rye yield based on a long-term series of multi-environment trials from 1983/1985 to 2016, associated with genotypic, spatial, and climatic covariates, revealed a decrease in the relative yield stability for wheat but an increase for rye [14]. In fact, winter rye turned out to be the most stable among four major European crops grown between 1955 and 2008 in a long-term fertilization experiment located in Germany [15]. These results mirror the strong adaptation potential of rye that refers to its unique mating system and that thrives in the continuous adaptation of this overlooked cereal [16] to a changing environment. To conclude, the cultivation of rye addresses emerging challenges of food security associated with climate change. However, the ever-changing demands of farmers, consumers, and the environment require an innovative strategy for selective matings to improve the performance of rye in a much shorter time as compared to natural selection and the production of phenotypes expressing traits beyond fitness to the environment in which rye is cultivated.

## 3. Hybrid Breeding, the Cutting-Edge Technology for a Systematic Improvement of Grain Yield in Rye

The systematic identification, management, and use of valuable natural diversity in outbreeding rye only became a feasible option following the establishment of hybrid breeding late in the 20th century. The rationale and main driver for hybrid breeding is the systematic exploitation of economic or standard heterosis that refers to the superiority of a hybrid over standard commercial check varieties. In rye, hybrid breeding is a success story in terms of the selection of favorable alleles for grain yield. In sharp contrast to the competitiveness of hybrid compared to line breeding in wheat [17], the key parameter that triggered the transition to hybrid rye was the high added value of hybrid varieties compared to open-pollinating cultivars [18], sufficient to cover the costs of seed production. An increase in productive tillers is a main factor for the strong genetic gain in grain yield of hybrid rye. The systematic exploitation of heterosis by hybrid breeding was initiated by Hartwig H. Geiger and Wolfgang Schnell at the University of Hohenheim around 1970 and the first hybrid rye cultivars were released in 1984 [19]. In 2022, the German Federal Plant Variety Office described 39 registered winter rye cultivars for grain use in the descriptive variety list [20]. Of these cultivars 76.9% are hybrids. The natural genetic diversity in rye was the fundamental basis to achieve a series of technological advances, over a century of breeding and research, that ultimately facilitated the establishment of hybrid breeding.

### 3.1. Trapping and Managing Genetic Diversity in Rye

**Figure 1:**
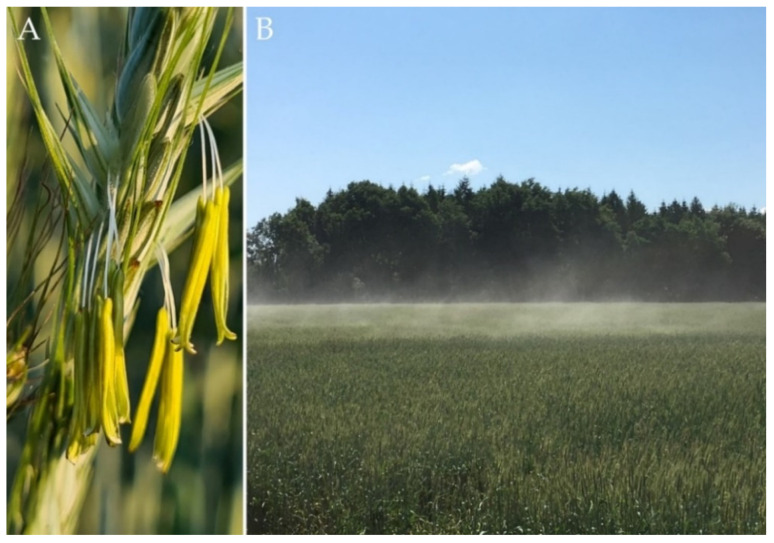
(**A**) Dehiscent anthers of a rye head. (**B**) Rye pollen dispersed by wind throughout a population.

**Figure 2:**
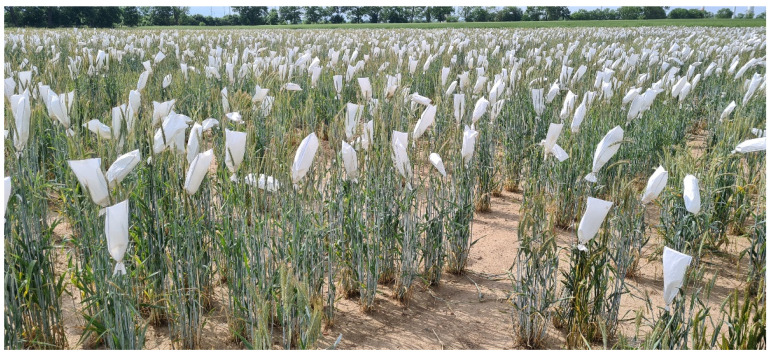
Production of elite rye inbred lines by bagging.

**Figure 3:**
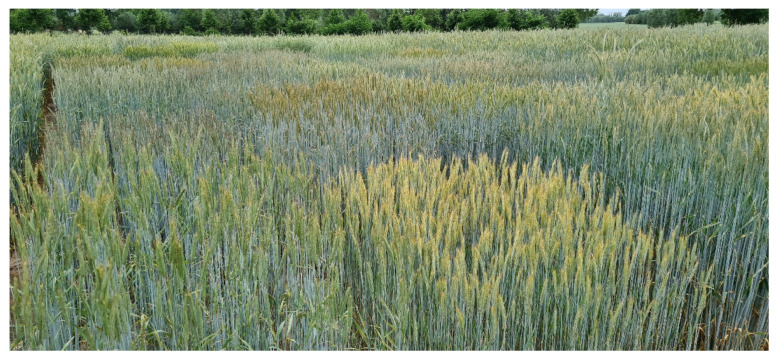
Inbred lines enable to assess and manage the genetic diversity of rye.

Cross-pollinating rye is unique among the small-grain cereals with respect to its reproduction biology (Figure 1). A single rye head produces approximately 4,200,000 pollen grains [21] that are dispersed by wind throughout a population. In open-pollinating rye populations, fertilization between genetically related individuals is prevented by a sophisticated bi-factorial cell-cell recognition system between the stigma and pollen grains [22]. As a consequence of this self-incompatibility (SI) system, the established seeds combine hereditary traits of both parents, and the resulting offspring constitute heterogenous cohorts of highly heterozygous genotypes. Species with functional SI such as rye have increased adaptive potential as a strong long-term evolutionary advantage and have been shown to diversify at a significantly higher rate than those without such genetic mechanisms promoting allogamy [23]. However, the SI system of rye constrains the development of purebred inbred lines with satisfying seed setting [24]. This challenge was a long-lasting topic of breeding research in rye and a reasonable number of inbred lines have been developed during the last century [25]. The ability to set self seed is highly heritable, and self offspring with high self-set were recovered from outbreeding rye populations by the Swedish botanist and geneticist Nils Heribert Nilsson [26] more than 100 years ago. Self-fertility mutations used in commercial hybrid rye breeding programs originate from material developed at two German locations, Müncheberg [27] and Gülzow [28]. The temporary conversion to an inbred plant is a high-throughput approach to capture and manage genetic diversity of rye that enables large-scale plant evaluation (Figure 2). Self-fertile rye inbred lines are highly versatile tools for testing the effect of genes and thousands of independent gene combinations on plant phenotypes in target environments of rye cultivation per year. Parent line development generally operates one or two stages of early-generation selection for line *per se* performance and is designed to measure alterations in highly heritable traits such as plant height, thousand-grain weight, and disease resistance with high sensitivity (Figure 3). Although double haploid (DH) technology has become a key tool to increase the speed and efficiency of plant improvement programs, the production of DHs in rye is yet not as efficient as for barley or wheat [29]. A recently published protocol reported progress in overcoming genotype dependency with respect to tissue culture responses of rye [30]. However, the development of parental inbred lines in commercial hybrid breeding programs is so far exclusively based on time-consuming, consecutive self-pollination (Figure 2). Indeed, the excellent dispersion of rye pollen by wind [31] requires careful control of pollination throughout the breeding process. 

Advances in deciphering the genetic code of *in vivo* haploid induction [32,33,34,35,36,37,38,39,40] and the recently published reference genome sequences for rye [41,42] offer the promising option to search for desired alleles in rye orthologs of the genes *MTL*/*ZmPLA1*/*NLD* or *cenh3.* This candidate gene approach might lead to an application of DH technology in rye to reduce the time required for producing parental lines by several years, as DH technology is known to simplify logistics [43]. The development of rye haploid inducer lines would make the breeding process more efficient and intuitive, and would substantially increase genetic gains in rye breeding programs to bring about higher-yielding, better-adapted cultivars at a faster pace. 

### 3.2. Unlocking Genetic Diversity for Selective Matings on a Large Scale

**Figure 4:**
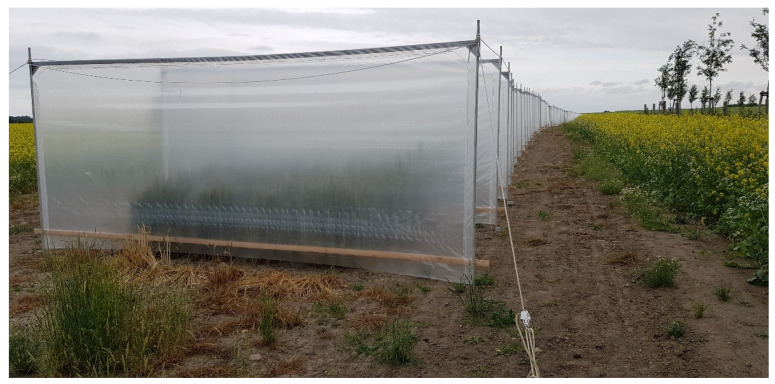
Production of experimental P-type CMS hybrids between foliar isolation walls.

To achieve the purpose of hybrid breeding, two phenomena are inevitably involved as a framework for exploiting genetic effects: inbreeding and heterosis. The reconstitution of heterozygosity by selective mating of inbred lines in the final step of a breeding cycle is an indispensable prerequisite to achieve the desired performance of rye in farmers’ fields. Although rye offers several cytoplasmic male sterility (CMS) systems [44,45,46,47,48] that enable selective mating, only the Pampa (P) cytoplasm from an Argentinean landrace [49] and later on the Gülzow (G) cytoplasm identified in adapted Central European rye populations [50,51] gained relevance in commercial hybrid rye breeding. Caused by the interaction between nuclear and mitochondrial genomes, CMS is a maternally inherited trait that causes dysfunctions in pollen and anther development [24]. The co-occurrence of females and hermaphrodites within rye is a relatively common sexual system in flowering plants and creates an evolutionary advantage. Empirical evidence has been reported that male sterile genotypes produced more flowers, set more fruits, and produced more seeds that were larger and germinated better than those of hermaphrodites from the same populations [52]. Such a fitness difference has been designated female advantage (FA) or female compensation [53]. A recently reported meta-analysis corroborated FA in seed number, which together with better seed germination, may explain maintenance of female plants within gynodioecious populations [54]. Indeed, CMS has significant positive effects on grain yield and thousand-grain weight in rye as well [55] and provides a natural, reliable, environmentally friendly, and cost-effective genetic system for hybrid seed production on a large scale (Figure 4). 

Both commercially used CMS systems differ in their impact on hybrid rye breeding. The G cytoplasm belongs to a plasmotype that is common in the Central European rye gene pool [56]. Its invasion in random mating rye populations indicates an obvious fitness advantage of this plasmotype in female function [57]. Once female frequency reached some threshold value, this probably led to a decrease in female seed production, which ultimately led females to be counter-selected [52]. As a consequence, restorer alleles for the G cytoplasm occur in high frequency [58]. This has a strong influence on practical applications. The unusual high linkage disequilibrium (LD) in the elite seed-parent pool [59] demonstrates that the development of male sterile seed-parent lines for breeding of G-type CMS hybrid rye is a challenging task due to a low frequency of reliable maintainer genotypes. This limitation hampers achieving the desired selection gain in the seed-parent pool of a breeding program. In contrast, the CMS-inducing P cytoplasm is easy to maintain and enables an unbiased capture and management of genetic diversity in the non-restorer pool [60]. However, the released G-type hybrid cultivars demonstrate the general feasibility of hybrid breeding using alternative hybridization systems in rye. The genetic diversity between the mitochondrial genomes of G- and P-type CMS hybrids [51,59,61,62,63] serves to reduce the potential genetic vulnerability of highly productive rye hybrids and asks for intensified research to unravel the genetic basis of the cyto-nuclear interaction of G-type CMS.

### 3.3. Fertility Restoration, Ergot Defense, and Yield Potential—The Challenging Triad

#### 3.3.1. The Genetics of Fertility Restoration in G-type CMS Is Coming of Age

In hybrid progenies from selective mating, the CMS effect of the seed-parent genotype needs to be counteracted by nuclear alleles from the pollen parent that restore pollen production. Such genetic factors, thus, are designated restorer-of-fertility (*Rf*) genes. Major *Rf* genes enabling grain production in CMS-based hybrids are available for both the G and the P cytoplasm, and have been mapped on the distal end of the long arm of chromosome 4R [62,64]. While male-fertility restoration by *Rfp* genes originating from non-adapted genetic resources is mainly monogenic dominant-inherited [64,65,66], the genetic architecture of male-fertility restoration of G-type hybrids reveals a more complex, oligogenic inheritance with a major gene that interacts with minor genes [51,62,67]. Recently, a genomic scan with the rye 600k SNP array contributed to further elucidate the genetic architecture of mito-nuclear interaction of G-type CMS and identified novel major G-type *Rf* genes on the short arm of chromosome 1RS and the long arm of chromosome 3R [61]. This progress should support the identification of non-restorer alleles for the development of reliable maintainer inbred lines with high general combining ability (GCA) to the pollen-parent pool for grain yield that is indispensable for achieving the desired selection gain in the seed-parent gene pool of rye breeding programs utilizing G-type CMS for hybrid seed production.

#### 3.3.2. Impact of Major *Rfp* Genes on Grain Yield in P-type CMS Hybrid Rye 

The development of PCR-based markers [65,68,69,70] resulted in an efficient implementation of dominant *Rfp* genes in hybrid rye breeding programs using the P cytoplasm as fertilization control system. These markers allow for a fast, reliable, and unbiased high-throughput tracking of introgressed segments carrying *Rfp* genes and, thus, increase the efficiency of foreground selection for male-fertility restoration. However, hybrids carrying an effective *Rfp* gene revealed a significant reduction in grain yield [71]. In a case study to disrupt the gene complex of the restorer gene Rfp1 with undesired genes that were transferred from the exotic donor into the elite material as well, a high-resolution genetic and physical map was used to support the marker-assisted identification of favorable recombinants at the Rfp1 locus in rye. The selection for individuals with a minimum of donor DNA was successful and the yield disadvantage associated with Rfp1 could be significantly reduced, but not completely eliminated [68]. As a consequence of this yield loss, a restricted integration of *Rfp* genes from weedy rye into elite pollinator synthetics resulting in a restorer index of ~50% is considered a viable practice [71,72]. However, CMS-based hybrids with an unsatisfactory restoration level and reduced pollen shedding are notably susceptible to ergot as the fungal spores have no competitors during the infection of the ovary [69]. 

#### 3.3.3. The Ergot Challenge

**Figure 5:**
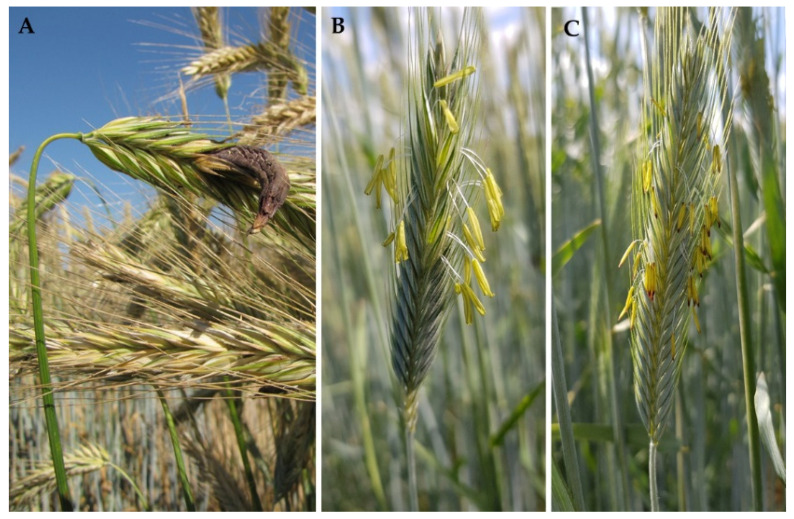
(**A**) *C. purpurea* infects the ovary and replaces the rye grain with a dark fungal body, the ergot sclerotium. (**B**) The gene *Rfp1* results in restoration of male fertility in P-type CMS rye hybrids and increases ergot defense. (**C**) Male sterile plants in P-type CMS rye hybrids with a restorer index of ~50% increase yield potential due to a female advantage but weaken the ergot defense.

Ergot is a disease of cereals and grasses caused by fungi in the genus *Claviceps*, with the chasmogamous rye as the principal economic host of *C. purpurea* (Fries) Tulasne, the species of greatest concern [73]. At anthesis, the open, non-fertilized florets enable ergot spores to access the stigma, mimic pollination, and infect ovaries of the host plant (Figure 5). 

Noteworthy in this context is a protective effect provided by ergot on cereal rye that probably acted against human exploitation, as heavily affected seed and grain samples have been, and still are, disposed of. This example of conditional host-pathogen interaction in which the consequence may fall on different parts of a continuum from parasitism to mutualism depending on the specific situation might explain why grasses are commonly susceptible and rather tolerant to ergot and no effective resistance mechanisms have evolved over time [74]. Indeed, though several *C. purpurea* virulence factors have been reported, RNAseq analysis of *in planta*-expressed fungal genes identified more than 400 highly expressed transcripts including an elevated frequency of genes encoding putative effectors that might be involved in repelling the fungal attack or interfering with host-defense interactions [75]. Although genetic variation for ergot resistance has been reported [76], specific rye defense reactions have yet not been described and the complexity of the system makes the identification and use of major effects triggered by single genes or large effect QTL unlikely. As a consequence, optimizing pollination by means of *Rf* genes is the most effective approach for minimizing ergot infestation in hybrid rye. Because of the toxicity of ergot alkaloids for humans and animals, the European Commission has significantly lowered the thresholds for ergot since 1 January 2022. Supplementary to the previous threshold of 0.05% ergot sclerotia in unprocessed rye, harvested grain may not contain more than 500 µg/kg of total ergot alkaloids in rye milling products for human consumption [77]. From 1 July 2024, these limits will be further reduced to 0.02% sclerotia and a maximum of 250 µg/kg alkaloids. The compliance of thresholds for ergot contamination in the harvest is, thus, critical for a reliable marketing of rye products. Therefore, the development of cultivars with strong ergot defense and high yield potential has a top priority to keep rye competitive in modern agricultural production systems.

#### 3.3.4. SMART Breeding to Counterbalance Linkage Drag Effects on Grain Yield 

**Figure 6:**
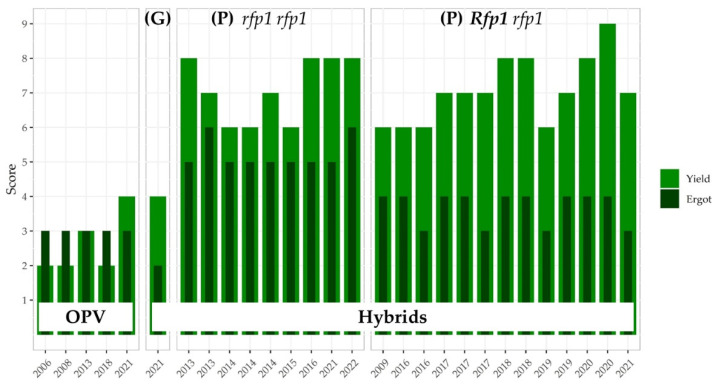
Ergot susceptibility and grain yield scores of the 30 entries of the German Descriptive Variety List 2022. Entries are represented by their year of release. The description of both traits is based on a 1–9 scale. A high figure indicates that the variety expresses the trait to a high degree, a low figure indicates that a variety shows the traits to a low degree, and 5 represents medium expression. (**G**) G-type CMS hybrid, (**P**) P-type CMS hybrids. *Rfp1*/*rfp1*: restorer/non-restorer allele at the *Rfp1* locus. Data source [20].

Susceptibility to ergot upon artificial inoculation is a trait assessed for registered rye varieties since 2008 in the descriptive variety list of the German Federal Plant Variety Office. The description of rye varieties listed in 2022 for their resistance and yield traits demonstrates genetic gain for ergot defense in highly productive hybrids already at the level of open-pollinating cultivars (Figure 6). However, the aforementioned undesired association between male fertility restoration and grain yield in rye hybrids could indeed yet not be overcome. Despite marker-assisted selection for a minimized donor-chromosome segment carrying *Rfp1* only [68], the development of varieties with a stronger ergot defense is still carried out at the expense of lower grain yield (Figure 6). In this regard it needs to be considered that *Rfp1* originates from a weedy rye population from the Fertile Crescent [64,65,70,78]. The observed yield penalty in rye can largely be attributed to a reduced thousand-grain weight [71]. This result is consistent with the observation of a 90% greater individual seed mass of cereal and pulse crops as compared to their progenitors from the Fertile Crescent [79]. Paternal effects such as that observed for *Rfp1* in rye have been reported influencing seed development through male gametogenesis or post-fertilization, due to factors deposited by the sperm cells in the egg and/or central cells, as well as by genomic imprinting [80]. Recently, modified heterotic responses by parent-of-origin effects on seed size have been reported in the model plant *Arabidopsis thaliana,* with a much larger influence of the paternal genome on F_1_ seed size than previously appreciated [80]. Notably, significant natural variation in the extent of the heterosis effects on F_1_ seed size has been observed in this study as well. These results from fundamental research have implications for breeding research on the seed biology of hybrid rye and open new avenues for genetic approaches to compensate for paternal contributions to seed size in rye hybrids with strong ergot defense. 

Our steadily growing knowledge of the genetic code of grain size and weight control [81,82] and known major genes governing thousand-grain weight in rye [25,60] offer a chance to develop genome-based precision breeding strategies and to counterbalance the linkage drag effects of non-adapted *Rfp* genes while simultaneously minimising the costs of restoration in terms of grain yield. Particularly promising for this purpose are genes that cause parent-of-origin effects on F_1_ seed size such as *MET1*, the primary maintenance DNA methyltransferase in *Arabidopsis thaliana.* Crosses between Arabidopsis genotypes carrying a complete loss-of-function and the wild-type *MET1* allele revealed that hypomethylated maternal genomes result in significantly larger F_1_ seeds [83]. In rye, the Cytosine-specific methyltransferase *SECCE2Rv1G0093370* [41] shares 55% identity at the amino acid level to *MET1*. In total, 6 SNP markers on the rye 600k SNP-array [84] provide a starting point to assess the genetic diversity of *SECCE2Rv1G0093370* between elite inbred lines and germplasm collections. Subsequently, an ingenious cross-validation strategy [60] allows for precise estimation of phenotypic effects of defined SNP alleles at candidate genes such as *SECCE2Rv1G0093370*. Superior SNP alleles leading to hypomethylated maternal genomes as measured by significantly larger F_1_ seeds are attractive means to offset the undesired paternal inherited effects of *Rfp1*.

This drafted option for selection with markers and advanced reproductive technology (SMART) breeding [85] in rye exemplifies the value of the recently published rye reference genome sequences [41,42] as invaluable tools to promote the genetic improvement of rye in terms of high yield potential and minimized risk of ergot infestation. 

#### 3.3.5. Linkage Drag Constrains the Ergot Defense of Hybrid Rye

**Figure 7:**
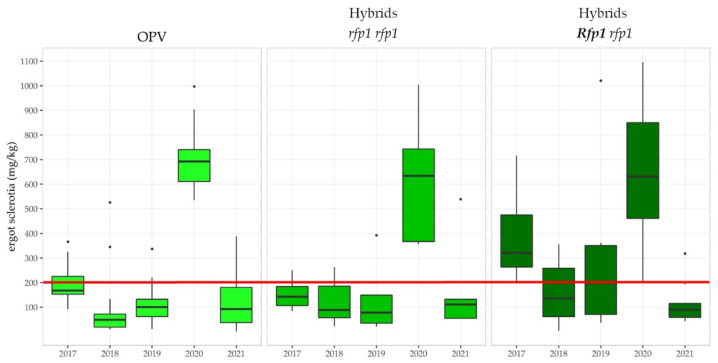
Ergot severity in rye upon natural infection detected in samples from open-pollinated (OPV) and hybrid cultivars merchandized without (*rfp1 rfp1*) and with (*Rfp1 rfp1*) the restorer gene *Rfp1* across 54 environments (location x year combinations). The number of environments varied between 8 (2019) and 15 (2020). The threshold of 200 mg ergot sclerotia/kg harvest valid from 1 July 2024 is indicated in red. Data source [89].

It needs to be emphasized that rainy weather at flowering time reduces pollen shedding and pollen movement. A suggested restorer index of ~50% [71,72] is a tribute to the undesired impact of major restorer genes such as *Rfp1* on grain yield but may result in insufficient quantities of pollen to combat the fungus adequately in farmers’ fields, as wet pollen agglutinates and distributes over short distances only. In fact, multi-environmental data on natural ergot infection provided by the Polish Research Centre for Cultivar Testing (COBORU) confirm this assumption (Figure 7). Favorable infection conditions resulted in ergot contamination of the harvest beyond the threshold that will be valid from 1 July 2024, in each of the 31 varieties assessed in 2020. The interquartile ranges are reasonably greater in hybrids compared to open-pollinated cultivars (OPV), which refers to the current heterogeneity of P-type CMS hybrids in terms of reproductive traits. The median ergot severity of all hybrids in 2020 is only slightly below the median of OPVs. Notably, the median ergot severity of hybrids traded as *Rfp1* carriers [72] was greater in 2017, 2018, and 2019 as compared to hybrids without this restorer gene, the latter of which were evaluated without a blend of population rye. More precisely, in the period from 2017 to 2021 the level of ergot sclerotia in unprocessed rye was above the upcoming threshold of 0.2 g/kg in 83%, 40%, 42%, 100% and 20% of the evaluated *Rfp1* hybrids. Remarkably, among 13 cultivars, including two *Rfp1*-carrying hybrids as well as four hybrids without *Rfp1*, which were assessed in all 54 environments, the average ergot severity was below the novel threshold only in two varieties released without *Rfp1.* According to the German descriptive variety list, these two hybrids were rated with a medium expression of ergot susceptibility, but with the hybrid released in 2016 (Figure 6) counting among the eight cultivars with very high yield potential. To conclude, this comprehensive multi-environmental dataset contravenes observations of ergot severity upon natural infections in eight other locations [72]. As a consequence, this extended assessment enables an open, evidence-based, and balanced discussion of the current strategy to use *Rfp1* for ergot defense in rye. Obviously, the genetic progress in ergot defense registered by artificial inoculation and a complex field design [86] in German trials assessing the Value for Cultivation and Use (VCU), the mandatory variety testing system for agricultural crops in the European Union, does not mirror the quality and performance standards required to combat the fungus adequately under natural infection conditions in farmers’ fields. The causes of this discrepancy are currently unclear and require further research. For instance, a strong selection for chasmogamous spikes with large and exposed stigmas enabling a rapid fertilization and maximum seed setting is probably underprivileged under the enforced artificial inoculation as compared to a more closed, cleistogamous-like spike morphology. The currently exercised ergot susceptibility assessment in German VCU trials, thus, could represent genetic differences in spike morphology as an ergot defense strategy rather than the natural competition between rye pollen and fungal spores. Such a critical function of floral traits conferring increased resistance by cleistogamous flowers and/or a short flowering period have been reported for the wheat/*Fusarium* [87] and the oat/*Ustilago* [88] pathosystems, respectively. Another reason for the distinguished ergot defense pattern between hybrid cultivars might refer to different selection strategies in the pollinator pool. In hybrids traded with *Rfp1*, selection against adapted restorer genes from European rye germplasm in the pollinator pool likely has contributed to weakening ergot defense upon natural infection, as, in hybrids with a restorer index of ~50% [71,72], the frequency of male sterile plants is very high. The driving force for this selection strategy refers to an increased selection efficiency for strong pollen-fertility restoration by *Rfp1* [72].

As a consequence of the illustrated strong environmental impact on the response of rye to ergot, and with respect to the relevance of this trait for the precautionary health protection of consumers, assessment of ergot defense in statutory VCU trials already in the first year of evaluation appears reasonable. Furthermore, the current methodological efforts and the associated costs suggest for an amendment of the procedures to further optimize the rating of a candidate variety’s performance in terms of ergot defense relative to that of National List varieties. Even more important and despite the two proven cultivars that enable minimising ergot in hybrid rye, varieties should be developed with a restorer index of 100%, i.e. restoration of male fertility in every single plant, in order to comprehensively mitigate the risk of ergot infection in rye. This strategy is the key in supporting short-distance pollen distribution even under unfavorable weather conditions at flowering, as hybrid rye – in contrast to open-pollinating varieties—is able to set seeds upon self-pollination, just as with wheat or barley. The G-type CMS hybrid released in 2021 (Figure 6) outperforms all other released hybrid and random-mating cultivars in terms of ergot defense rating and serves as a model for the suggested approach. This particular hybrid illustrates how the long-lasting ergot challenge in rye can be overcome by hybrid breeding to comprehensively comply with stringent thresholds of ergot contamination in the harvest. For this purpose, the natural genetic diversity at the *Rfp* locus and less pronounced linkage drag effects of *Rfp2* and *Rfp4* on grain yield compared to *Rfp1* [90] open attractive options for the breeding of ergot-resistant and toxin-free rye hybrids with high yield potential. 

### 3.4. Heterotic Groups, the Enigma of Complementing Components for Yield Improvement

Hybrid breeding enables the specific use of heterosis in agriculture. Heterosis is also known as hybrid vigour and describes the biological phenomenon of a genetically superior genotype by combining its parents’ values. Hybrids are characterized by a particularly effective light interception machinery beginning with seedling vigour and including homeostasis of photosynthesis over a broad range of environmental conditions as the basis for a strong source [91]. Transcriptional profiling in rice revealed that hybrid breeding enables an increased activity of enzymes involved in carbon fixation pathways and net photosynthetic rate [92]. In both maize and Arabidopsis, expression of various genes for photosynthesis has been observed above mid-parent levels [93,94,95]. In maize, a positive correlation between grain yield and the net photosynthetic rate upon the year of release of hybrid cultivars has been reported [96]. Hybrid rye breeding, thus, is a key technology for increasing grain productivity by enhancing photosynthesis, the primary determinant of grain yield [97].

The single most important element in implementing hybrid breeding is the recognition of a high-yielding heterotic pattern [98]. Indeed, two factors that have contributed critically to the success of hybrid rye breeding are the classification of elite germplasm into heterotic groups and the identification of a high-yielding heterotic pattern. A heterotic group is a set of genetically related genotypes that show similar hybrid performance when crossed with individuals from another genetically distinct germplasm group [99]. In rye, the two heterotic groups ‘Petkus’ and ‘Carsten’ reveal a heterotic pattern by expressing pronounced performance of their offspring in grain yield [100]. Rye breeders empirically grouped the parental lines with higher grain yield, high grain weight, and better lodging resistance into the female heterotic group termed ‘Petkus’. The corresponding parents with large spikes and good seed set but lower *per se* performance were classified into the male heterotic group termed ‘Carsten’. Both gene pools have been developed in Germany. At the end of the 19th century Ferdinand von Lochow III succeeded in breeding a considerably undemanding but very productive type of rye on predominantly diluvial sandy soils and during harsh winters at the manor Petkus located south of Berlin [101]. The original ‘Petkus’ winter rye did not fail even under better climate and soil conditions, but was able to adapt well and brought high, reliable grain yields. At the beginning of the 20th century, Rudolf Carsten started his rye breeding program in Bad Schwartau close to the Baltic Sea using Prof. Heinrich-Roggen as a stock [102]. The pedigree of Carsten’s rye traces back to a Scandinavian landrace that revealed a very short, compact head [103]. Recently, flow cytometry revealed significant differences in the haploid genome size of ‘Petkus’ and ‘Carsten’ rye [41]. Whether the genome size difference between both heterotic groups refers to a large extent of gene-order and structural variations just as in maize [104], and its potential impact on the heterotic pattern deserves further research.

## 4. A Recurrent Selection Scheme to Enrich Desirable Alleles for Grain Yield

**Figure 8:**
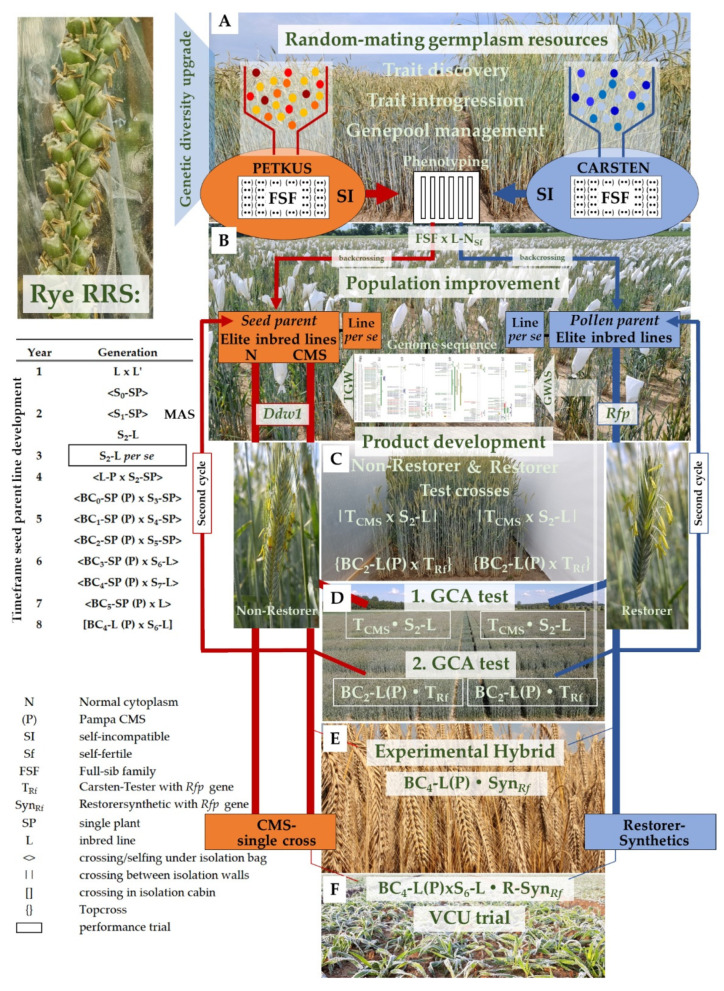
Flow chart illustrating a reciprocal recurrent selection (RRS) scheme in rye. (**A**) Random mating germplasm resources serve as an upgrade in genetic diversity. The coloured circles represent different alleles that are systematically captured and introgressed in both heterotic gene pools. (**B**) Improvement of the breeding population is based on self-fertile rye inbred lines as highly versatile tools for testing the effect of genes and gene combinations on plant phenotypes. Knowledge how the genome builds, maintains, and operates rye gained for example by Genome-wide Association Studies (GWAS) allows to increase selection efficiency for traits such as thousand-grain weight (TGW) by marker-assisted selection (MAS), as already realized for *Dominant dwarf 1* (*Ddw1*) and Restorer-of-fertility genes for Pampa CMS (*Rfp*). (**C**) Hybrid breeding enables to select crossing partners on a rational basis. Testcrosses are established between isolation walls or as topcrosses using cytoplasmic male sterility as a condition for large scale seed production, as CMS tester (T_CMS_) genotypes of rye are unable to produce functional pollen. (**D**) The general combining ability (GCA) of parental genotypes is estimated based on testcross performance in four (1. GCA test) to ten (2. GCA test) target environments of rye cultivation. (**E**) The generation and evaluation of restorer synthetics (Syn*_Rf_*) from crosses between pollinator inbred lines is necessary to neutralize the inbreeding depression on pollen production that rye is suffering as a cross-pollinating crop. (**F**) Certified seed is produced in a technical mixture of 95% CMS single-cross seed parent and 5% restorer synthetic. The thickness of lines mirrors a decreasing number of entries as a result of phenotypic selection.

The frequency of favorable alleles for grain yield in modern hybrid rye breeding programs is improved based on reciprocal recurrent selection (RRS) [105]. RRS is a cyclic selection that aims to enhance the breeding population as a whole in grain yield through crossing and recombination. It involves the systematic selection made on the basis of testcross performance, intermating, and recombination of selected plants, and requires enhanced hands-on time to develop elite rye inbred lines by selfing (Figure 8B). In rye RRS, the heterozygosity that is temporarily lost due to selfing is recovered by intermating of selected inbred lines (Figure 8C). Initially, the standard scheme of seed-parent line development required the time-consuming development of CMS analogues of S_4_ and S_6_ lines in backcross generations BC_1_ and BC_2_, respectively, for the development of testcross seeds and elite two-line restorer synthetics as testers [106]. A greater gain in efficiency is now achieved by male-sterile testers from the pollinator gene pool for producing testcross seed of the candidate seed-parent lines before first CMS analogues (BC_1_, BC_2_) of them become available. The current breeding scheme is two generations shorter than the standard scheme [106], and thus speeds up breeding progress. Analogous, pollinator line development is based using elite CMS testers of the seed-parent pool. As a consequence, inbred genotypes with superior line *per se* performance are selected from the base population and crossed to two heterozygous testers representing the ‘Carsten’ and ‘Petkus’ pool. The testcrosses are subsequently evaluated in 3–4 target environments with respect to their yield performance to identify the GCA of the selected genotypes (Figure 8D). Based on a selection intensity of approximately 20%, CMS analogues of S_6_ lines in backcross generations 

BC_2_ are used to establish further testcrosses to achieve robust performance data on yield potential, stability, and adaptation with the second GCA test in 8 to 10 highly divergent environments in terms of soil fertility and precipitation (Figure 8D). This approach ensures that rye hybrids are able to adjust to many environmental conditions, as has been demonstrated by multi-environmental field phenotyping of 658 intra- as well as interpool testcrosses [107]. Furthermore, the evaluation of such defined rye genotypes in large yield trials with sufficient ecological and biological replications, and genome-wide association studies [60] provide the desired framework that allows deciphering of the genetic architecture of complex inherited traits such as grain yield (Figure 8B,D). Identifying relevant genes shaping the genetic diversity in agronomic traits of rye is a crucial step for making previously hidden genetic variation accessible to genetic studies and the breeding of rye. According to Geiger [106], the progeny of at least 30 divergent genotypes with good GCA are intermated in all possible combinations to adequately restrict the loss of genetic variation due to random drift and selection. Their crossed seeds constitute the improved population for further selection in the next cycle. RRS supports maintaining genetic variability in the population due to repeated intermating of selected genotypes. The continuous improvement of the breeding population enables the use of yield trials for RRS (Figure 8D) as ‘early testing’ trials for cultivar development (Figure 8F). Hence, the best lines can be extracted from each cycle of selection for building up hybrids that are superior to those from previous cycles [106].

Progress in high-density SNP genotyping of rye [84] enabled the implementation of genomic selection as a breeding strategy for improving the hybrid performance of the pollen-parent pool [108]. Recurrent genomic selection promises to shorten the length of selection cycles and increase selection gain in hybrid rye [109]. However, the impact of genotype-by-environment interactions on recurrent genomic selection emphasizes the necessity to develop robust genome-wide predictive equations [110]. In rye, the separation of line development from product development is indispensable when genomic selection is integrated as a tool in the seed-parent pool to render the development of CMS analogs of selected genotypes a feasible task. As a consequence, cost-effective schemes for product development that are integrated into the line development program [106] need to be reorganized [109]. The reorganization of a hybrid rye breeding program asks for further research to better understand the balance between risks, that could have a negative impact on the success of a breeding program, e.g. by reducing phenotypic information and increasing demands for the glasshouse, nursery, and genotyping resources, and rewards in terms of an expected higher genetic gain. In contrast, progress in establishing a speed breeding protocol for winter cereals [111] has immediate potential to accelerate the improvement of winter hybrid rye by optimizing the inevitable vernalization protocol. This technical advance offers a shortening of the generation time for developing CMS analogs of seed-parent lines which is a limiting factor and currently extends the timeline of hybrid rye breeding (Figure 8B).

## 5. Random-Mating Rye Populations, the Genetic Diversity Booster

The strong selection on GCA to the opposite pool results in a steadily increasing genetic differentiation between both heterotic pools in rye. Estimated based on randomly sampled, genome-wide molecular markers, the calculated fixation index *F_ST_* = 0.229 [84], *F_ST_* = 0.332 [59], and *F_ST_* = 0.185 [60] emphasized the efficient design of hybrid rye breeding programs for the systematic and steady improvement of grain yield performance. However, this approach and genetic drift result in a decrease in genetic diversity during the breeding process. Furthermore, beyond both heterotic groups, and just like in maize [112], the structure of elite hybrid rye breeding populations is characterized by unique sub-heterotic groups that are largely isolated between commercial rye breeding programs. As a consequence, and in contrast to wheat or barley, the use of elite germplasm from competitive programs is impossible in hybrid rye breeding. Thus, harnessing the allelic diversity of complex traits such as grain yield from genetic resources is essential for meeting future demands on rye production (Figure 8A). 

Indeed, random-mating populations supercharge the genetic diversity in rye. Plant breeding comprises the science and art to combine favorable alleles in a novel and unique manner. For this purpose, genetic recombination, i.e. the exchange of genetic material between parental genotypes, is of central importance because it allows plant breeders to identify and select novel combinations of favorable alleles that differ from those found in either parent for the genetic improvement of crops [113,114]. However, the accumulation of crossover breakpoints in second cycle rye recombinant inbred lines, i.e., from crosses among elite inbreds within heterotic pools, is limited as each generation of inbreeding renders the recombining chromosomes more similar to one another, so that meiosis ceases to generate new recombinant haplotypes. In contrast, intra-genic linkage-disequilibrium decays rapidly in population rye, within approximately 520 bp on average [115], referring to a high recombination frequency as the only primary mechanism to break down the non-random assortment of alleles at different loci. Random-mating populations, thus, accelerate the rye breeding process through modulating meiotic recombination, the key natural process that generates genetic diversity. Furthermore, the long-lasting and ongoing cultivation of random-mating rye populations *in situ* results in a directional selection for stable grain production in ever changing environments. Random-mating rye populations serve as a reservoir of diversity that holds readily available but untapped genetic diversity for potential climate-responsive traits and their use in hybrid rye breeding. To conclude, germplasm resources are of paramount importance for upgrading genetic diversity in hybrid rye breeding programs.

Random-mating populations can be introduced into hybrid breeding programs with relative ease (Figure 8A). Their genetic improvement is based on a full-sib family recurrent selection scheme [7] that is a crucial pillar within hybrid rye breeding to target and further develop meiotic recombination. Importantly, testcrosses of germplasm resources with both heterotic pools have to be established and evaluated, as deficiencies made in the correct classification of germplasm resources to the established heterotic pools will negatively affect the heterotic pattern and, as a consequence, the competitiveness of a breeding program. The integration of novel diversity in elite breeding populations is achieved by backcrossing to elite inbred lines which eliminates deleterious recessive alleles. Recently, a novel sampling strategy has been recommended for the improvement of elite germplasm of allogamous crops such as rye. Instead of maximizing diversity with few individuals sampled from many landraces covering a wide range of geographic regions, high-density SNP genotyping enables a systematic identification of novel alleles adapted to a specific set of environments and to the genetic background of a target elite breeding pool from few pre-selected landraces [116]. The recently developed 600k SNP array [84] enables adaption of this approach to rye. Likewise, a previously suggested breeding scheme [117] could be customized to revert the self-fertility mutations used in elite inbred lines to the wild-type. SNP markers residing at both self-incompatibility loci *S* and *Z* [24] allow for the desired tracking of the wild-type allele during the backcrossing of individual elite inbred lines. Such advanced germplasm resources will tune the rates of recombination between elite alleles and enable their dynamic management by cultivation in ecologically contrasting locations, where the populations can evolve over time under an environment-specific selection pressure. This adjustment of evolutionary plant breeding [118] to rye is the most reliable approach to achieve broad adaptation and deliver varieties that can still perform during poor seasons [119]. The suggested approach may be particularly relevant for the pollinator gene pool, as the probability of finding populations that are genetically diverse from the ‘Petkus’ pool is reduced due to the dominant role of the ‘Petkus’ rye as an ancestor of many random mating populations globally [120].

## 6. Approaching Rye Genes Controlling Grain Yield

**Figure 9:**
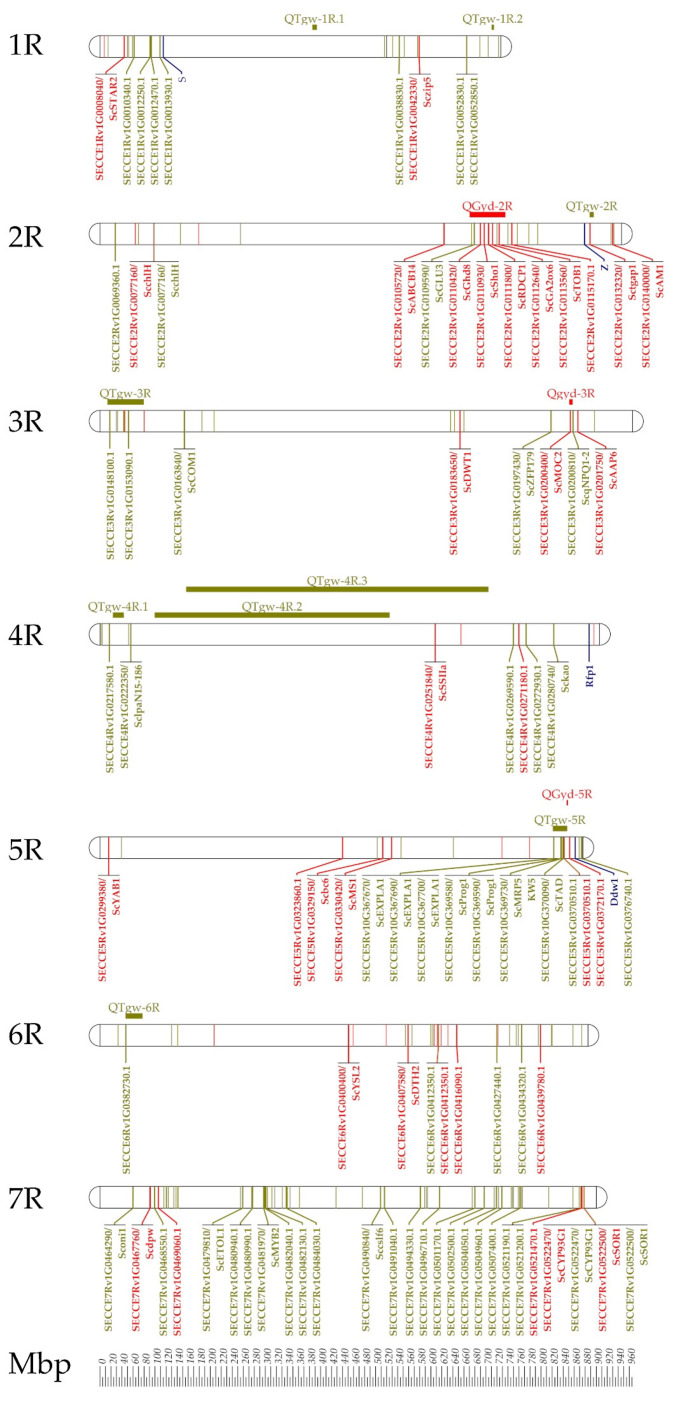
Physical position of cross-validated SNPs in protein coding sequences and intergenic regions of the ‘Lo7’ genome assembly detected in the GWAS for grain yield (red) and thousand-grain weight (green). The positions of both self-incompatibility loci, *S* and *Z*, the restorer-of-fertility locus *Rfp1* depicting the rye’s unique reproduction biology, and the GA-sensitive dwarfing gene *Ddw1* are given as well. For Lo7 orthologs of cloned rice QTL the corresponding rice gene symbols were adapted to rye. The positions of the markers in the Lo7 physical map are given in Mbp. The horizontal bars and QTL symbols indicate the position of grain yield (*QGyd*), and thousand-grain weight (*QTgw*). Data source: [60].

Grain yield in rye is complex inherited and determined by three quantitative traits: the number of productive tillers, the number of grains per spike, and the thousand-grain weight (TGW). Recently published reference genome sequences [41,42] and hybrid breeding provide powerful tools for investigating the genetic and molecular basis of these quantitative traits. A recently published genome-wide association study [60] extends the first QTL study in rye hybrids [121] and serves as a foundation experiment by providing insights into the genetic architecture of grain yield for rye improvement (Figure 9). The comprehensive phenotyping of 526 genetically diverse experimental hybrids in replicated field trials in up to 19 environments for grain yield, grain quality, and agronomic traits identified morphological adjustment of plant height and tiller number as drought stress responses conferring yield stability of rye. Cross-validation of individual marker-trait associations identified 38 single nucleotide polymorphisms (SNPs) associated with grain yield including 13 SNPs representing protein-coding sequences that are physically mapped in the Lo7 reference genome sequence [60]. The comparative analysis between rye and rice for similar or homologous traits identified a conserved genetic architecture for agronomic traits that served as cross-species validation. In total, 19 and 14 SNPs in protein-coding genes of the ‘Lo7’ assembly associated with GYD and TGW were identified as orthologs of cloned rice QTL [60]. These results demonstrate that integration and advancement of fundamental and applied breeding and research from rice [122,123,124,125] in the rye reference genome sequences [41,42] supports our understanding how the genome builds, maintains, and operates rye.

Knowledge of genomic regions controlling complex traits is the key to our understanding of mechanisms behind yield architecture and to using them in marker-assisted crop improvement programs. The novel catalog of SNP variations associated with yield and grain weight holds potential for the clear-cut and systematic genetic improvement of yield potential in rye hybrids with strong ergot defense. Hybrid breeding offers a pragmatic strategy to evaluate the influence of single genomic loci on grain yield in rye. Progenies segregating for superior (S) or inferior (I) SNP alleles can be used to establish near-isogenic genotype bulks (NIB) of homozygous elite F3 lines of the seed-parent pool, that will serve as pollinators in crosses with male-sterile single-cross testers from the opposite pool between isolation walls [60]. In the first instance, allelic variation within a candidate gene that controls sufficient and consistent (across genetic backgrounds) increase in yield potential delivers the desired tools to offset the costs of restoration in hybrid rye. Validated SNPs could serve as predictive markers that support selection decisions about numerous aspects of rye’s yield performance. The reference genome sequences of rye [41,42] allow for the continuous integration of further progress in deciphering the molecular basis of grain yield [126,127] and to extend the compilation of SNPs associated with grain yield in rye. To further increase the value of this approach, large effect SNPs correlating with grain yield might stimulate research to the deciphering of gene functions as a prerequisite for the development of functional markers. With the release of two high-quality genome assemblies [41,42], the systematic search for SNPs located in coding sequences and within a gene’s regulatory sequences, changing the function, timing, location, or level of gene expression, offers promising perspectives for this purpose.

Although the development of perfect markers that address the functional sequence polymorphism of a gene governing grain yield would be the consistent final step, it needs to be considered that the necessary functional characterization of a candidate gene by transposon tagging, transformation for overexpression, knockdown analysis, or genetic modification based on novel plant breeding techniques is still in its infancy, i.e. not available or published in rye. The extension of the molecular toolbox available for rye by sophisticated technologies probably is a long-term perspective. In contrast, the proposed International Functional Genomics Project on Rye (IFGPR) for the development of highly replicable rye genetic stocks in the public domain [25] appears feasible for providing indispensable support in the short term.

## 7. Novel Ideotypes to Improve the Yield Potential of Rye

The introgression of major dwarfing genes for increasing yield potential is a proven concept in wheat and rice [128,129,130,131]. In rye, in particular the gene *Dominant dwarf 1* (*Ddw1*), identified five decades ago by Vladimir Kobylyansky in the germplasm collection at the N.I. Vavilov Institute of Plant Genetic Resources [132] and initially described as *Estestvennyj Mutant 1* (*EM1*, russ.: natural mutant 1), has been used in breeding of open pollinating cultivars for the genetic improvement of plant height [25], a quantitative inherited trait with complex genetic architecture in rye [60,108,133,134,135]. However, this approach is hampered by increased frequencies of tall plants, as heterozygous semidwarf genotypes carrying the undesired recessive wild-type allele are difficult to detect within the seed multiplication process of open-pollinating populations [136,137,138,139]. Efficient DNA-profiling with PCR-based markers [136,140,141] offers the possibility to overcome this challenge. In contrast to random-mating rye populations, *Ddw1* has not been used in hybrid breeding programs due to theoretical considerations [7,133,134,135]. As a consequence, winter rye was by far the tallest among five cereal crops in a recent survey of German variety trials on the long-term breeding progress of yield and yield-related traits [142]. 

**Figure 10:**
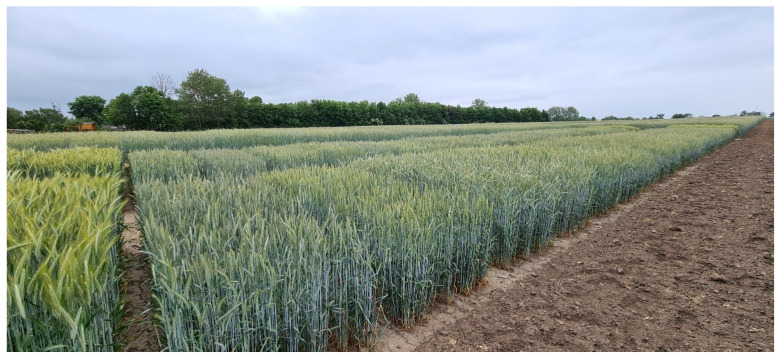
Survey on the first 48 gibberellin-sensitive semidwarf P-type CMS rye hybrids.

In order to evaluate the potential of *Ddw1* in hybrid rye, novel markers [141] increased target-specific selection efficiency for the introgression of *Ddw1* into the seed-parent pool. Homozygous semidwarf BC_3_S_2_ single-cross testers enabled the development of semidwarf P-type hybrids in 2020 for the first time ever. Subsequently conducted performance trials in European and Canadian target environments for rye cultivation revealed that the semidwarfs were distinct from existing conventional hybrid rye varieties and expressed the genetically reduced plant height uniformly (Figure 10). All prototypes met the requirements for approval as a variety on the basis of the Seed Trade Act, as hybrid breeding overcomes the described challenge in breeding semidwarf random-mating populations and keeps the genetically reduced plant height stable. Indeed, in a period of eight years subsequent to the first crosses and extensive phenotypic evaluation, the first semidwarf P-type CMS hybrids were applied for national listing at the German Federal Plant Variety Office in August 2022. Inspired by the pioneering work of the plant breeder and Nobel Peace Prize winner Norman Borlaug, these novel plant breeding innovations are advancing the Green Revolution in small grain cereals by integrating a gibberellin-sensitive dwarfing gene for genetic growth regulation and the systematic exploitation of heterosis by hybrid breeding. Semidwarf rye hybrids illustrate the effective use of marker-assisted selection for tapping valuable genetic diversity provided by plant genetic resources, for the precise and rapid development of new crop varieties.

**Figure 11:**
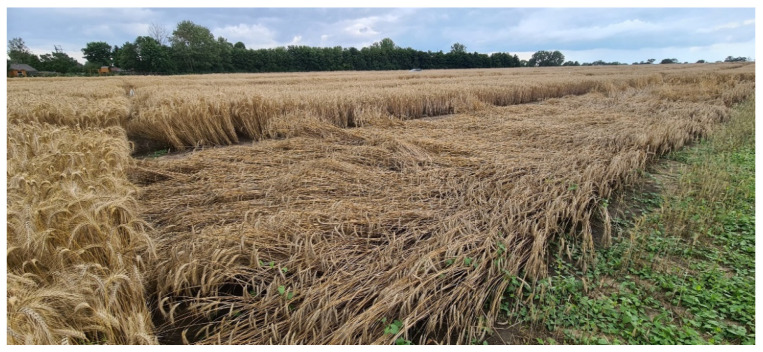
Genetic growth regulation by the major dwarfing gene *Ddw1* confers pronounced lodging resistance. The displacement of culms from an upright position of the near-isogenic conventional experimental hybrids in the foreground subsequent to extreme precipitation of 200 L/m^2^ could not be prevented although they were treated with chemical plant growth regulators.

Keeping the benefits of conventional rye, the cultivation of these novel ideotypes with a revised plant architecture, high yield potential, and improved ergot defense will promote more sustainable agricultural systems in the EU and other rye producing regions. In contrast to their near isogenic conventional full-sibs treated with chemical plant growth regulators (PGRs), the semidwarfs revealed a pronounced lodging resistance as a result of their shorter and sturdier stems (Figure 11). In view of extreme precipitation with up to 200 L/m^2^ at the beginning of July 2021 at several trial locations, this result is proof of concept that genetic growth regulation using dominant dwarfing genes for protection from yield loss is feasible in rye and confirms the desired core functionality of semidwarf hybrids in a central agronomic trait. Semidwarf rye cultivars, thus, hold the potential to displace PGRs as a currently indispensable component of plant growth management in rye cultivation for achieving maximum yield, and assuring harvestability. Semidwarf rye passes farmers’ challenging responsibility for growth regulation with PGRs at the correct time of application, and dose rate, as well as the crop’s growing conditions, back to the plant. Furthermore, the genetic lodging resistance enables farmers to explore higher planting densities with semidwarf hybrids, providing the potential to produce more rye on finite arable land. Semidwarf hybrids improve harvestability of rye as well. A crop in a good stand enhances harvest efficiency by increasing the speed of the harvest while reducing the consumption of fossile fuels. In cereal crop rotations, semidwarf rye improves straw management which is of central importance for the perfect establishment of the subsequent crop. Since residues of especially one active substance, chlormequat, are sometimes found in detectable levels in cereal food products, there is an ongoing discussion in the Cereal Food Industry to limit the possible presence of PGRs in cereal products [143]. Semidwarf rye delivers a sustainable solution to promote human health by providing new and healthier grain products devoid of any potential acute or chronic dietary exposure to PGR residues. Taken altogether, the non-chemical control method of plant height in semidwarf rye is a novel, key plant breeding innovation to support the main objectives of the European Green Deal by reducing the overall use of chemical pesticides while ensuring the resilience and security of food supply in the EU. 

*Ddw1* belongs to the group of gibberellin (GA)-sensitive dwarfing genes [144]. Noteworthy in this context, it is becoming increasingly evident that the GA class of plant hormones is of pivotal relevance in the response of plants to abiotic stress [145]. Positive effects on grain yield, lodging tolerance as well as drought tolerance in tef (*Eragrostis tef* (Zucc.) Trotter) and finger millet (*Eleusine coracana* Gaertn) have been reported as a result of chemically induced GA deficiency [146]. In rice, induced mutants of the GA deactivation gene GA2-oxidase 6 (GA2ox6) moderately reduced GA concentration and reprogrammed transcriptional networks, leading to reduced plant height, more productive tillers, an expanded root system, higher water use efficiency and photosynthesis rate, and elevated abiotic and biotic stress tolerance [147]. In accordance with these observations the analysis of the Arabidopsis NAC-like GIBBERELLIN SUPPRESSING FACTOR (GSF) showed a novel function in the regulation of gibberellin biosynthesis [148]. The ectopic expression of GSF lacking a transmembrane domain (GSF-TM) caused a dwarf phenotype, which was correlated with the upregulation of *GA2ox2*/*6* and an increased drought tolerance compared to the wild-type plants. In semidwarf rye the gene *ScGA2ox12* co-segregating with the GA-sensitive dwarfing gene *Ddw1* is up-regulated as well [141], supporting the value of this natural gene variant for rye breeding. The described progress in our understanding of the importance of GA-homeostasis for drought stress tolerance, thus, implies that the established semidwarf rye hybrids most likely confer both, lodging and drought tolerance in rye, as the *Ddw1* mutant alters the GA content in a favourable manner. Notably, the integration of the *Ddw1* target interval in the wheat reference genome sequence indicated perfect micro-colinearity between the *Ddw1* locus and a 831 kb segment on chromosome 5A, which resides inside of a 11.21 Mb interval carrying the GA-sensitive dwarfing gene *Rht12* in wheat [141]. The knowledge gained on semidwarf rye hybrids argues for the utilization of recently generated *Rht12* overgrowth mutants with a range of intermediate height phenotypes taller than the original *Rht12* stock [149] as novel options for the development of climate-smart wheat. 

Concerns have been raised about a potentially negative impact of genetically reduced culm length in rye on photosynthesis and yield performance [7,133,134,135,142]. In fact, in a biparental population, negative pleiotropic effects of *Ddw1* on the line *per se* performance in thousand-grain weight and flowering time have been reported [150]. However, performance trials assessing effects and side-effects of *Ddw1* on the testcross performance of genetically divers rye hybrids, and in a reasonable number of environments, were lacking until recently. Indeed, extensive phenotyping of conventional experimental hybrids representing a high level of genetic diversity in two years with contrasting precipitation patterns improved our understanding of rye’s adaptation to drought stress [60]. This comprehensive phenotypic dataset did not compromise the use of major dwarfing genes such as *Ddw1* as an option to tap currently unused potential for optimized dry matter allocation to the rye grain. However, it may be misleading to transfer knowledge from conventional to semidwarf genotypes without empirical data. For instance, in semidwarf *Ddw1* genotypes the head has proven to be supplied with assimilates essentially via the flag leaf (F) and F-1 [151], in contrast to conventional rye genotypes where the tall culm serves as the main assimilation organ [7]. The comprehensive evaluation of semidwarf hybrids in performance trials conducted in 2021 and 2022 provides the missing link and valuable information to rate the effects and side effects of *Ddw1* on the performance of rye in target environments of rye cultivation. The enhanced implementation of *Ddw1* in hybrid rye breeding serves as a viable example of breeders’ options to target allelic combinations of genes controlling phenology and plant architecture. In this way, further known major genes such as *VRN*, *PPD*, *EPS* [152], and genes associated with winter field survival in rye [153] are worth characterizing as fundamental contributors for the adaptation to specific rye-growing regions and farm management systems. 

## 8. Conclusions

Despite current achievements, major challenges in rye production remain for advancing rye from its current status as a healthy minor cereal to a trend-setting crop with increasing relevance for grain production in a changing climate. Therefore, improved breeding efforts are strategically important for enhancing the competitiveness of rye in agricultural production systems. In rye, hybrid breeding serves as a cutting edge technology to select crossing partners on a rational basis for breeding improved cultivars with different complex but complementary traits to achieve cumulative gene action for grain yield and yield stability. The identification of traits that may allow rye to capture its most limiting resources more efficiently receives strong support from two high-quality rye genome assemblies [41,42] and progress in physiological breeding [154]. Major genes determining plant height count among the candidates with high priority for genetic improvement requirements in orphan crops, which are generally only grown and valued locally or regionally, just as with rye. As described in the present review, semidwarf rye supports three areas of fundamental importance for addressing food production and human demographic trends and their associated challenges, namely: increased integration into production systems; improving the processability of crop products; and reducing farm labour requirements [155]. The development of gibberellin-sensitive semidwarf P-type CMS hybrid rye provides the desired innovation in grain productivity improvement that is considered as a fundamental requirement in plant breeding driving increased production diversification with orphan crops [155]. Thus, the successful development of semidwarf rye increases the range of biological and low risk alternatives for sustainable production of healthy grain on the market and delivers on a key element of the European Green Deal to restore Europe’s nature by 2050 by increasing crop diversity in agricultural ecosystems. The recent implementation of *Ddw1* in a practical hybrid rye breeding program is paradigmatic for specific genes and adaptive alleles that govern important agronomic traits in rye. The promising performance in target environments of rye cultivation triggers an enhanced development of semidwarf hybrids and may initiate a new era of physiological rye breeding that aims to raise the yield potential of rye closer to its biological limit. Indeed, the rich genetic diversity of rye, together with a sophisticated bulked segregant phenotyping strategy of testcross performance in multi-environmental field trials, argue for a stronger utilization of rye in research directed to the identification of valuable alleles controlling yield and yield components for Triticeae improvement programs. For this purpose, further investment in resequencing is promising for an improved understanding of the genetics of adaptive traits in rye.

Hybrid breeding had obvious advantages and positive side effects in rye. In contrast to many valuable crops with a relatively small acreage that became orphans for which breeding was no longer carried out [156], hybrid rye, as with quinoa, chickpea and pigeonpea [157], serves as a further example of orphan crops with obvious potential for reducing the dependence of the world food system on a limited number of crops. The benefits of hybrid rye to farmers are increased yield potential, and strong lodging resistance in semidwarf rye. Furthermore, hybrid breeding offers scientific and genetic advancement to combat the Achilles’ heel of open-pollinated rye cultivars, the ergot fungus, effectively. Hybrid rye breeding supports agricultural biodiversity for a resilient food and farming system with continuously improved hybrid cultivars, to the benefit of farmers, consumers, and the environment. Notably, hybrid breeding represents a technology with the potential to strengthen global food and nutrition security [17]. This cutting-edge technology it is not restricted to multinational breeding companies, as demonstrated by the hybrid rye breeding program of a medium-sized enterprise described in this review. However, for its long-term success, unrestricted access to new technologies, knowledge, and native traits is indispensable to safeguard the existing diversity of innovative plant breeding programs with their proven benefits for genetic gain in yield and yield stability of small-grain cereals. The development of gibberellin-sensitive semidwarf hybrids based on predictive breeding technologies, leading to new cereal phenotypes and varieties with improvements in plant health, protection, production, and resilience, is a further example of the innovation potential of small- and medium-sized enterprises in the field of cutting-edge plant breeding research for a sustainable bioeconomy.

## Data Availability

Not applicable.
